# Adolescent binge alcohol exposure accelerates Alzheimer’s disease-associated basal forebrain neuropathology through proinflammatory HMGB1 signaling

**DOI:** 10.3389/fnagi.2025.1531628

**Published:** 2025-02-19

**Authors:** Rachael P. Fisher, Lindsay Matheny, Sarrah Ankeny, Liya Qin, Leon G. Coleman, Ryan P. Vetreno

**Affiliations:** ^1^Bowles Center for Alcohol Studies, School of Medicine, University of North Carolina at Chapel Hill, Chapel Hill, NC, United States; ^2^Department of Pharmacology, School of Medicine, University of North Carolina at Chapel Hill, Chapel Hill, NC, United States; ^3^Department of Psychiatry, School of Medicine, University of North Carolina at Chapel Hill, Chapel Hill, NC, United States

**Keywords:** ethanol, neuroinflammation, microglia, cholinergic neurons, HMGB1

## Abstract

Human studies suggest that heavy alcohol use may be an etiological factor contributing to the development of Alzheimer’s disease (AD) neuropathology. Both alcohol use disorder (AUD) and AD share common underlying neuropathology, including proinflammatory high-mobility group box 1 (HMGB1)-mediated neuroimmune signaling and basal forebrain cholinergic neuron degeneration. Adolescent onset of binge drinking represents a significant risk factor for later development of an AUD, and accumulating evidence suggests that adolescent initiation of heavy alcohol use induces HMGB1 signaling and causes degeneration of the basal forebrain cholinergic system that persists into adulthood. However, it is unknown whether adolescent binge drinking confers increased risk for later development of AD-associated neuropathology through persistent induction of proinflammatory HMGB1 neuroimmune signaling. To investigate this question, we first (Experiment 1) assessed AD-associated neuropathology in the post-mortem human basal forebrain of individuals with AUD and an adolescent age of drinking onset relative to age-matched moderate drinking controls (CONs). In Experiment 2, we treated non-transgenic and 5xFAD male and female mice, which overexpress both mutant human APP and PS1, with adolescent intermittent ethanol (AIE; 5.0 g/kg, i.g. 2-days on/2-days off; postnatal day [P]30 – P55), and assessed AD-associated neuropathology in the adult (P100) basal forebrain. In Experiment 3, 5xFAD female mice received AIE treatment followed by glycyrrhizic acid (150 mg/L), an HMGB1 inhibitor, in drinking water from P56 to P100, and basal forebrain tissue was collected on P100 for assessment of AD-associated neuropathology. In the post-mortem human AUD basal forebrain (Experiment 1), we report upregulation of *Hmgb1* and the HMGB1 receptors *Rage* and *Tlr4* as well as microglial activation and increased intraneuronal Aβ_1–42_ accumulation in association with reduced cholinergic neuron marker expression (ChAT). In the 5xFAD mouse model (Experiment 2), AIE accelerated AD-associated induction of *Hmgb1* proinflammatory neuroimmune genes, microglial activation, and reductions of ChAT+ basal forebrain cholinergic neurons in the adult female, but not male, basal forebrain. In Experiment 3, post-AIE treatment with glycyrrhizic acid rescued the AIE-induced acceleration of AD-associated increases in proinflammatory HMGB1 neuroimmune signaling, microglial activation, and persistent reductions of basal forebrain cholinergic neurons in adult 5xFAD female mice. Together, these findings suggest that adolescent binge ethanol exposure may represent an underappreciated etiological factor contributing to onset of AD-associated neuropathology in adulthood through HMGB1- mediated neuroimmune signaling.

## 1 Introduction

Adolescence, which spans 10–24 years of age in humans (Sawyer et al., 2018) (rodents = approximately 21–60 days of age), is a conserved neurodevelopmental period. Across mammalian species, adolescence is characterized by refinement of brain neurotransmitter systems (Shoval et al., 2014), including the cholinergic system of the basal forebrain (Semba, 2004; Coleman et al., 2011), that parallels the transition from child to adult characteristic behaviors (Spear, 2009). The increased neuroplasticity that characterizes the adolescent brain also increases its vulnerability to environmental insults that can impart long-lasting changes in adult neurobiology. Alcohol binge drinking is common during adolescence. Approximately 20% of individuals ages 16–17 and 34% of individuals ages 18–25 engaging in binge drinking in the United States in the past month (NSDUH, 2020), and approximately 10% of high school seniors endorse high-intensity binge drinking consisting of 10+ drinks and 5.6% reporting 15+ drinks in a single drinking session (Patrick et al., 2013). Evidence suggests that the developing adolescent brain is more sensitive to alcohol-induced damage than the adult brain (Crews et al., 2000; Crews et al., 2007). Adolescent binge drinking is associated with lasting consequences that persist into adulthood, including increased risk of developing an alcohol use disorder (AUD), persistent deficits in cognition, disruption of brain maturation, and long-lasting induction of proinflammatory neuroimmune signaling (Grant and Dawson, 1998; Grant et al., 2001; Vetreno and Crews, 2012; Vetreno et al., 2013; Vetreno and Crews, 2015, 2018; Macht et al., 2021; Vetreno et al., 2021). The unique vulnerability of the adolescent brain to long-lasting changes in neurobiology suggest that adolescent binge drinking could increase the risk for development of neuropsychiatric disorders later in life. Indeed, adolescent intermittent ethanol (AIE) exposure, a preclinical rodent model of human adolescent binge drinking, causes progressive degeneration of basal forebrain cholinergic neurons that persists into adulthood (Vetreno and Crews, 2018) similar to cholinergic deficits observed in Alzheimer’s disease (AD) (Lehericy et al., 1993; Beach et al., 2000; Stepanichev et al., 2017; Hampel et al., 2018). Emerging epidemiological studies implicate heavy alcohol use and AUD as risk factors for later development of dementia and AD-associated neuropathology (Fratiglioni et al., 1993; Mukamal et al., 2003; Langballe et al., 2015; Schwarzinger et al., 2018) with additional studies suggesting a link between heavy alcohol use early in life with increased risk for AD in late adulthood (Langballe et al., 2015). More recently, preclinical rodent models of adolescent binge ethanol exposure have reported an exacerbation of AD-associated neuropathology in adult AD mouse models (Barnett et al., 2022). Thus, adolescent binge drinking may represent an emerging, but underappreciated, epidemiological risk factor contributing to the onset of AD-associated neuropathology in adulthood.

Alzheimer’s disease is a progressive neurodegenerative disease characterized by a gradual decline in cognitive ability that is typically diagnosed in individuals over the age of 65 (Atri, 2019). While a diagnosis of AD is predominantly determined post-mortem by the presence of amyloid (Aβ) plaques and tau neurofibrillary tangles in the brain, the etiology of AD remains largely unknown although several risk factors have been posited, including sex and lifestyle factors such as smoking, excessive alcohol use, and diabetes (Schwarzinger et al., 2018). Intriguingly, adolescent binge drinking, AUD, and AD share several pathological commonalities, including induction of proinflammatory neuroimmune signaling and degeneration of basal forebrain cholinergic neurons. For instance, studies in adult AIE-treated rats as well as in human AUD and AD studies report reductions of basal forebrain cholinergic neurons and increased expression of proinflammatory Toll-like receptor 4 (TLR4), receptor for advanced glycation end-products (RAGE), and the endogenous TLR4/RAGE cytokine-like agonist high-mobility group box 1 (HMGB1) (Arendt et al., 1983; Vetreno et al., 2014; Heneka et al., 2015; Crews et al., 2019; Paudel et al., 2020; Vetreno et al., 2021). HMGB1 is a unique endogenous cytokine-like nuclear protein constitutively expressed by all nucleated cells of the brain. It is released from the nucleus in response to alcohol and other stressors wherein it acts as an endogenous ligand at pattern recognition receptors (e.g., RAGE, TLR4), leading to persistent induction of proinflammatory signaling. HMGB1 activation of TLR4, RAGE, and other receptors leads to nuclear translocation of NF-κB and subsequent induction of neuroimmune signaling molecules, contributing to complex proinflammatory signaling (Mayfield et al., 2013; Crews et al., 2017). Importantly, human plasma levels of HMGB1 are increased in adolescent binge drinkers and post-mortem human brain of individuals with AUD and an adolescent age of drinking onset as well as in the cerebrospinal fluid of individuals with AD (Fujita et al., 2016; Orio et al., 2018; Vetreno et al., 2021). The persistent AIE-induced reductions of basal forebrain cholinergic neurons is accompanied by long-lasting increases of HMGB1, TLR4, RAGE, phosphorylated (activated) NF-κB p65 (pNF-κB p65), and downstream proinflammatory neuroimmune genes in the basal forebrain and throughout the adult brain (Vetreno and Crews, 2012; Vetreno et al., 2013; Vetreno and Crews, 2018; Vetreno et al., 2018; Crews et al., 2021; Crews et al., 2023). Degeneration of the basal forebrain cholinergic system is reported to occur early in life in individuals who later develop AD, and heavy alcohol consumption (i.e., ≥ 8 drinks/week) exacerbates cognitive decline in individuals with early AD (Grothe et al., 2012; Heymann et al., 2016). Thus, adolescent binge ethanol exposure may confer increased risk for later development of AD-associated neuropathology through persistent induction of proinflammatory HMGB1 neuroimmune signaling.

Given the commonalities in neuropathology observed across models of human adolescent binge ethanol exposure and AD, we sought to test the hypothesis that adolescent binge drinking increases AD-associated neuropathology in adulthood through proinflammatory HMGB1 signaling in the post-mortem human AUD basal forebrain and in the 5xFAD mouse model of AD. In the post-mortem human AUD basal forebrain, we report induction of proinflammatory HMGB1-RAGE/TLR4 and evidence of microglial activation as well as increased intraneuronal Aβ_1–42_ in association with reduced cholinergic neuron marker expression (ChAT). In the 5xFAD mouse model, we report that AIE accelerated AD-associated induction of HMGB1 proinflammatory neuroimmune genes, microglial activation, and reductions of ChAT+ basal forebrain cholinergic neurons in the adult female, but not male, basal forebrain. Finally, post-AIE treatment with the selective HMGB1 inhibitor glycyrrhizic acid rescued AIE-induced acceleration of AD-associated increases in proinflammatory HMGB1 neuroimmune signaling, microglial activation, and persistent reductions of basal forebrain cholinergic neurons in adult 5xFAD female mice. Together, these data suggest that adolescent binge ethanol exposure may be an etiological factor contributing to onset of AD-associated neuropathology in the adult basal forebrain through HMGB1-neuroimmune signaling.

## 2 Materials and methods

### 2.1 Post-mortem human basal forebrain tissue samples

Post-mortem human basal forebrain paraffin-embedded sections and frozen tissue samples from moderate drinking controls (CONs) and individuals with AUD (*n* = 8/group) were obtained from the New South Wales Brain Tissue Resource Centre [NSW BTRC (Ethnics Committee Approval Number: X11-0107)] at the University of Sydney {supported by the National Health and Medical Research Council of Australia-Schizophrenia Research Institute and the National Institute of Alcohol Abuse and Alcoholism [NIH (NIAAA) R24AA012725]}. Individual information was collected through personal interviews, next-of-kin interviews, and medical records, and is presented in Table 1. The NSW BTRC donor program uses a pre-mortem consent program inviting members of the community to donate their post-mortem brain tissue to neuroscience research, allowing collation and study of individuals with a wide variety of clinical histories of AUD and alcohol drinking. Since establishing an accurate alcohol drinking history and age of drinking onset is critical, trained clinical nurses and psychologists from the NSW BTRC performed extensive interviews with the human volunteers and their families. Alcohol drinking history and age of drinking onset was determined through personal interviews with the volunteers as well as medical records and next-of-kin interviews. In cases where the age of drinking onset was unclear, an age of 25 was recorded by the NSW BTRC (Sheedy et al., 2008). Individuals with AUD reported an average age of drinking onset of 17 (± 1.0) years of age, which was compared to age-matched CONs whose average age of drinking onset was 24 (± 1.0) years of age. It is noteworthy that 100% of individuals with AUD were able to recall their age of drinking onset whereas only one moderate drinking CON individual was able to provide accurate information. Moderate drinking was defined as consuming 2 or fewer alcoholic beverages per day (> 20 g/day). Only individuals with AUD uncomplicated by liver cirrhosis and/or nutritional deficiencies were included in the present study. All psychiatric and AUD diagnoses were confirmed using the Diagnostic Instrument for Brain Studies that complies with the Diagnostic and Statistical Manual of Mental Disorders (Dedova et al., 2009).

**TABLE 1:** Demographics of human post-mortem moderate drinking control (CON) and alcohol use disorder (AUD) individuals.

Classification	Age of death	Brain weight (g)	PMI (h)	Clinical cause of death	Brain pH	RIN	Age of drinking onset	Years drinking	Lifetime alcohol consumption (kg)
CON	48	1,330	24	Cardiac	6.7	6.9	25	23	16.8
CON	44	1,220	50	Cardiac	6.6	7.1	25	19	27.7
CON	43	1,400	66	Pneumonia	6.2	Unknown	25	18	Unknown
CON	60	1,420	28	Cardiac	6.8	8	25	35	Unknown
CON	24	1,490	43	Cardiac	6.3	6.2	20	4	14.6
CON	50	1,426	30	Cardiac	6.4	7.5	25	Unknown	Unknown
CON	62	1,430	46	Cardiac	7.0	8.8	25	7	5.1
CON	50	1,596	40	Cardiac	6.9	8.6	25	25	18.3
AUD	51	1,240	27	GI hemorrhage	5.6	Unknown	17	34	Unknown
AUD	50	1,520	17	Cardiac	6.3	7	18	32	2,452.8
AUD	44	1,360	15	Cardiac	6.5	7.9	20	10	638.8
AUD	42	1,400	41	Toxicity	6.5	8	18	24	1,471.7
AUD	45	1,580	19	Respiratory	6.6	7.9	15	29	1,799.5
AUD	49	1,600	44	Cardiac	6.4	6.4	16	33	1,011.8
AUD	49	1,420	16	Cardiac	6.2	6.2	14	35	613.2
AUD	61	1,340	24	Cardiac	6.9	8.3	17	44	5,621

Age of drinking onset is significantly different [*t*(14) = −8.2, *p* < 0.001] between CON (24 ± 1) and AUD individuals (17 ± 1). Lifetime alcohol consumption [CON: 17 kg ± 4, AUD: 1,944 kg ± 661; *t*(7) = 2.7, *p* = 0.030, Welch’s *t*-test], years drinking [CON: 19 ± 4, AUD: 30 ± 4; *t*(13) = 2.2, *p* = 0.051], and post-mortem interval [PMI; CON: 41 h ± 5, AUD: 25 h ± 4; *t*(14) = 2.5, *p* = 0.027] are significantly different between groups. No differences were observed regarding age of death (*p* = 0.791), brain weight (*p* = 0.758), brain pH (*p* = 0.196), and RNA integrity number (RIN; *p* = 0.683).

### 2.2 Human immunohistochemistry

Paraffin-embedded post-mortem human basal forebrain sections were deparaffinized, washed in phosphate-buffered saline (PBS), and antigen retrieval performed by incubation in Citra solution (BioGenex, San Ramon, CA, USA) for 1 h at 70°C. Following incubation in blocking solution (MP Biomedicals, Solon, OH, USA), slides were incubated in a primary antibody solution for 24 h at 4°C. Primary antibodies, dilutions, and validation information are presented in Table 2. Slides were incubated for 1 h in biotinylated secondary antibody (1:200; Vector Laboratories, Burlingame, CA, USA) and then for 1 h in avidin-biotin complex solution (Vector Laboratories). The chromogen nickel-enhanced diaminobenzidine (Sigma-Aldrich, St. Louis, MO, USA) was used to visualize immunoreactivity. Slides were dehydrated and cover-slipped. Negative control for non-specific binding was conducted employing the above-mentioned procedures with omission of the antibody.

**TABLE 2:** Primary antibodies used for immunohistochemistry.

Species	Antibody	Isotype (IgG)	Dilution	Company, catalog number	Validation
Human	Aβ1-42	Rabbit	1:250	Invitrogen, 700254	ELISA, IHC (Invitrogen)
Human	CD68	Mouse	1:40	Dako, M0814	IHC (Dako)
Human	ChAT	Goat	1:200	Millipore, AB144P	Blocking antibody (Current study)
Human	Iba-1	Rabbit	1:400	Wako, 019-19741	IHC (Wako)
Mouse	Aβ1-42	Rabbit	1:500	Invitrogen, 700254	ELISA, IHC (Invitrogen)
Mouse	CD68	Rabbit	1:80	Abcam, ab283654	KO (Abcam)
Mouse	ChAT	Goat	1:200	Millipore, AB144P	Blocking antibody (Current study)
Mouse	HMGB1	Rabbit	1:1000	Abcam, ab18256	KO (Abcam)
Mouse	Iba-1	Rabbit	1:500	Wako, 019-19741	IHC (Wako)
Mouse	pNFκBp65	Rabbit	1:2000	Abcam, ab86299	WB (Abcam)
Mouse	RAGE	Rabbit	1:1000	Abcam, ab3611	IHC, WB (Abcam)

### 2.3 Human immunofluorescence and colocalization analysis

Paraffin-embedded post-mortem human basal forebrain sections were deparaffinized, washed in Tris-buffered saline (TBS), and antigen retrieval performed by incubation in Citra solution (BioGenex) for 1 h at 70°C. Slides were incubated in blocking solution (MP Biomedicals) followed by incubation for 24 h at 4°C in a primary antibody solution consisting of blocking solution with ChAT in combination with an antibody against Aβ1-42 (see Table 2). Slides were washed in TBS and incubated for 1 h at room temperature with the appropriate Alexa Fluor secondary antibodies (1:1000; ThermoFisher Scientific, Waltham, MA, USA). Secondary-only negative controls were performed without primary antibody incubation. Slides were cover-slipped using Prolong Gold Anti-fade mounting media (Life Technologies, Carlsbad, CA, USA). Immunofluorescent images were obtained using a Nikon DS-Ri2 scope (Nikon Inc., Melville, NY, USA) and colocalization quantified using NIS Elements AR46 analysis software (Nikon Inc.).

### 2.4 Human RNA isolation and reverse transcription PCR (RTPCR)

Total RNA was extracted from frozen human basal forebrain tissue samples from CON and AUD subjects by homogenization in TRI reagent (Sigma-Aldrich) following the single-step method of RNA isolation (Chomczynski and Sacchi, 2006). RNA quality and concentration was determined using a NanoDrop 1000 (ThermoFisher Scientific). Total RNA was reverse transcribed as previously described (Vetreno et al., 2018). RTPCR was run using a Bio-Rad CFX system (Bio-Rad Laboratories, Hercules, CA, USA). SYBR Green PCR Master Mix (Life Technologies) was used for RTPCR. RTPCR was run with an initial activation for 10 min at 95°C followed by 40 cycles of denaturation (95°C, 15 s), annealing/extension (57–58°C, 1 min), and melt curve. The primer sequences are presented in Table 3. Differences in primer expression between groups are expressed as cycle time (Ct) values normalized with *βactin*, and relative differences between groups calculated and expressed as the percent difference relative to CONs.

**TABLE 3:** List of primers for RTPCR.

Species	Primer	Forward	Reverse
Human	*App*	5′-GTC CAG AAT GGG AAG TGG GA-3′	5′-CAC TGC ATG TCT CTT TGG CG-3′
Human	*Hmgb1*	5′-GGA GAT CCT AAG AAG CCG AGA-3′	5′-CAT GGT CTT CCA CCT CTC TGA-3′
Human	*Rage*	5’-CTA CCG AGT CCG TGT CTA CCA-3’	5’-CAT CCA AGT GCC AGC TAA GAG-3’
Human	*Tlr4*	5′-TAC AAA ATC CCC GAC AAC CTC-3′	5′-AGC CAC CAG CTT CTG TAA ACT-3′
Human	*βactin*	5′-GCA TGG GTC AGA AGG ATT CCT-3′	5′-TCG TCC CAG TTG GTG ACG AT -3′
Mouse	*Hmgb1*	5′-CAG GAG TGG CTT TTG TCC CTC-3′	5′-CCG CAG TTT CCT ATC GCT TT-3′
Mouse	*Il6*	5′-GGC CTT CCC TAC TTC ACA AG-3′	5′-ATT TCC ACG ATT TCC CAG AG-3′
Mouse	*Rage*	5′-GAA GGC TCT GTG GGT GAG TC-3′	5′-CCG CTT CCT CTG ACT GAT TC-3′
Mouse	*Tlr4*	5′-GCC TTT CAG GGA ATT AAG CTC-3′	5′-AGA TCA ACC GAT GGA CGT GTA A -3′
Mouse	*Tlr6*	5′-ATT AAA GTC TGG GGC TGT TTG C-3′	5′-TTT GTT CTT GGT GGC AGG TCT-3′
Mouse	*Tlr7*	5′-ATG TGG ACA CGG AAG AGA CAA-3′	5′-GGT AAG GGT AAG ATT GGT GGT G-3′
Mouse	*Tlr8*	5′-TTG CTG ATG CTA AAG CGA CC-3′	5′-TCT TTC TTG GAA GGC TCT TGC T-3′
Mouse	*Tnf*α	5′-CAA GCC TGT AGC CCA CGT C-3′	5′-ACA AGG TAC AAC CCA TCG GC-3′
Mouse	*18s*	5′-GGT AAC CCG TTG AAC CCC AT-3′	5′-CAA CGC AAG CTT ATG ACC CG-3′
Mouse	*βactin*	5′-GGC TGT ATT CCC CTC CAT CG-3′	5′-CCA GTT GGT AAC AAT GCC ATG T-3′

### 2.5 Animals

Hemizygous female 5xFAD mice (Jackson Laboratories, MMRRC Stock #034848-JAX) overexpressing the mutant human amyloid beta (A4) precursor protein 695 (APP), the Swedish (K670N, M671L), Florida (I716V), and London (V717I) familial Alzheimer’s disease (FAD) mutations, and the human PS1 harboring two FAD mutations (M146L, L286V) were bred with wild type male C57BL/6J mice (Jackson Laboratories, Stock #000664). Male and female non-transgenic (Non-Tg) and 5xFAD mice bred and reared at the University of North Carolina at Chapel Hill were used in this study. On postnatal day (P)15, ear punches were collected for genotyping (Transnetyx, Cordova, TN, USA). Animals used for experimentation were weaned on P27 and randomly assigned to treatment conditions, and group-housed by sex and genotype (4–5 animals per cage) in standard cages in a temperature- and humidity-controlled vivarium on a 12 h/12 h light/dark cycle, and provided *ad libitum* access to food and water. This study was conducted in an AAALAC-accredited facility in strict accordance with NIH regulations for the care and use of animals in research. All experimental procedures reported in this study were approved by the Institutional Animal Care and Use Committee at the University of North Carolina at Chapel Hill and conducted in accordance with NIH regulations (Protocols 20-190 and 20-231). Two separate cohorts of animals were used in the current study with male and female Non-Tg and 5xFAD mice used in Experiment 1 and female 5xFAD mice used in Experiment 2.

### 2.6 Adolescent intermittent ethanol (AIE) model

Across experiments, adolescent male and female Non-Tg and 5xFAD mice were randomly assigned to AIE or water control (CON) conditions on P27. To minimize the impact of litter variables, no more than one subject from a given litter and sex was assigned to a single experimental condition. From P30 to P55, AIE subjects received a single daily intragastric (i.g.) administration of ethanol (5.0 g/kg, 25% EtOH, w/v) in the morning on a 2-day on/2-day off schedule for a total of 7 binge ethanol bouts, and CON subjects received comparable volumes of water on an identical schedule as previously described (Vetreno et al., 2020; Crews et al., 2021; Barnett et al., 2022; Macht et al., 2023). We have previously reported that this EtOH dosing regimen results in binge levels of intoxication producing peak blood ethanol concentrations (BECs) at 1 h of 280–300 mg/dL (Coleman et al., 2011; Coleman et al., 2014). This BEC is similar to blood alcohol levels attained by binge drinking human adolescents who were reported to intake approximately 13 drinks/episode, consistent with blood alcohol levels of 250–300 mg/dL (Deas et al., 2000; Jones and Holmgren, 2009). Body weights were assessed through AIE to the conclusion of experimentation.

**FIGURE 1:**
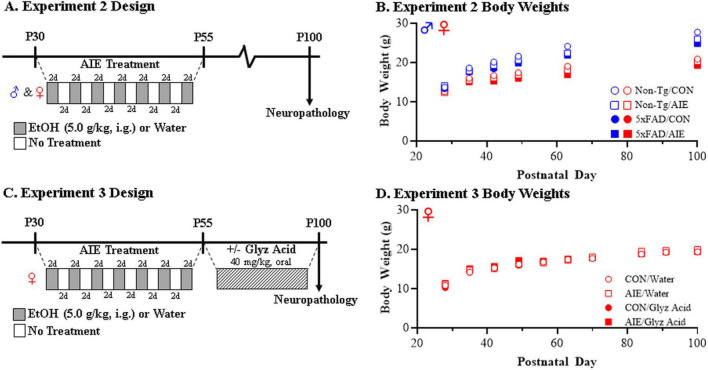
Experimental design for adolescent intermittent ethanol (AIE) experiments in mice. **(A)** Adolescent female and male non-transgenic (Non-Tg) and 5xFAD mice received either water gavage (i.g.) or ethanol (EtOH; 5.0 g/kg, i.g.) treatment on a two-day on/two-day off administration schedule from postnatal day (P)30 to P55. Following treatment conclusion, CON- and AIE-treated mice were left alone without intervention until sacrifice and tissue collection on P100. **(B)** Body weights of female and male CON- and AIE-treated Non-Tg and 5xFAD mice across treatment and maturation into adulthood. **(C)** Adolescent female 5xFAD mice received either water (i.g.) or EtOH (5.0 g/kg, i.g.) treatment on a two-day on/two-day off administration schedule from P30 to P55. Twenty-four hour following the conclusion of AIE treatment, CON- and AIE-treated mice received post-AIE treatment with either vehicle (water) or glycyrrhizic acid (Glyz Acid; 150 mg/L, P.O.) in water bottles from P56 until sacrifice and tissue collection on P100. **(D)** Body weights of female CON- and AIE-treated 5xFAD mice across treatment and adult vehicle and glycyrrhizic acid treatment. Data presented as mean ± SEM.

In Experiment 1, Non-Tg and 5xFAD male and female mice (*N* = 105; see Table 4 for subject numbers) received CON or AIE treatments and tissue was collected for analysis on P100 (see Figure 1A). All subjects in Experiment 1 evidenced dramatic body weight increases across experimentation (main effect of Age: *p* < 0.01; repeated measures ANOVA) with male subjects weighing more than female subjects (main effect of Sex: *p* < 0.01). Further, 5xFAD mice generally weighed slightly less than their Non-Tg littermates (main effect of Genotype: *p* < 0.01) and AIE treatment slightly decreased body weights relative to CONs (main effect of Treatment: *p* = 0.039) (see Figure 1B).

**TABLE 4:** Sample sizes per group in Experiment 1.

Condition	Cohort	*n* sizes
Non-Tg/CON	1	♂ = 7; ♀ = 7
	2	♂ = 7; ♀ = 6
Non-Tg/AIE	1	♂ = 6; ♀ = 6
	2	♂ = 7; ♀ = 7
5xFAD/CON	1	♂ = 7; ♀ = 7
	2	♂ = 7; ♀ = 7
5xFAD/AIE	1	♂ = 6; ♀ = 7
	2	♂ = 7; ♀ = 7

IHC (Cohort 1), PCR (Cohort 2).

In Experiment 2, female 5xFAD mice (*N* = 36; *n* = 8–10 subjects per treatment) received either CON or AIE treatment (i.e., P30–P55) followed by daily treatment with either glycyrrhizic acid (HMGB1 inhibitor; treatment protocol described below) or water vehicle beginning 24 h following the conclusion of AIE on P56 until tissue collection for analysis on P100 (see Figure 1C). While subjects in the glycyrrhizic acid reversal study evidenced dramatic body weight increases across experimentation (male effect of Age: *p* < 0.01), we did not observe an effect of AIE (main effect of Treatment: *p* = 0.34) or glycyrrhizic acid (main effect of Drug: *p* = 0.86) on body weight (see Figure 1D).

### 2.7 Glycyrrhizic acid treatment

Glycyrrhizic acid, which is a constituent of *Glycyrrhiza glabra* (licorice root) classified by the FDA as Generally Recognized As Safe as a food additive, is a potent, selective HMGB1 inhibitor that binds to and inactivates released HMGB1 (Mollica et al., 2007). To determine if glycyrrhizic acid reverses AIE-induced enhancement of AD-associated basal forebrain neuropathology, group-housed CON- and AIE-treated female 5xFAD mice received post-AIE treatment with glycyrrhizic acid (150 mg/L, P.O.; Sigma-Aldrich; Cat. #50531) or vehicle (water) in cage water bottles from P56 to P100. Prior to glycyrrhizic acid treatment, subjects were habituated to home cage water bottles from P51 to P56. Total liquid consumed was measured daily, and total liquid consumed per day in each cage was divided by the number of subjects within the cage to determine approximate consumption volumes. While AIE treatment did not affect overall water consumption (main effect of Treatment: *p* = 0.14), water consumption increased during habituation (main effect of Day: *p* < 0.01) (see Supplementary Figure 1A). During treatment, subjects in the glycyrrhizic acid condition consumed more liquid (main effect of Drug: *p* < 0.05) whereas liquid consumption did not differ as a function of time or treatment condition (see Supplementary Figure 1B). On average, subjects in the glycyrrhizic acid condition consumed an average of 4.0 mL/day with an approximate average consumption of 30.0 mg/kg of glycyrrhizic acid per day.

### 2.8 Perfusion and brain tissue preparation

Across experiments, subjects were sacrificed on P100 and brain tissue collected for assessment. For immunohistochemical studies, mice were deeply anesthetized with isoflurane (4.0%; Pivetal, Liberty, MO; Cat. #P151A) and transcardially perfused with 0.1 M PBS followed by 4.0% paraformaldehyde in PBS. Brains were excised and post-fixed in 4.0% paraformaldehyde for 24 h at 4°C followed by 4 d fixation in 30% sucrose solution. Coronal sections were cut (40 μm) on a sliding microtome (MICROM HM450; ThermoFisher Scientific), and sections sequentially collected into well plates and stored at −20°C in a cryoprotectant solution (30% glycol/30% ethylene glycol in PBS). For RTPCR studies, mice were deeply anesthetized with isoflurane and transcardially perfused with 0.1 M PBS, and basal forebrain tissue rapidly dissected, frozen in liquid nitrogen, and stored at −80°C.

### 2.9 Mouse immunohistochemistry

Free-floating basal forebrain tissue samples [every 6th section; approximate Bregma: 1.33–0.13 mm based on the atlas of Paxinos and Franklin (2019)] were washed in 0.1 M PBS, incubated in 0.6% H_2_O_2_ to inhibit endogenous peroxidases, and blocked with normal serum (MP Biomedicals). Sections were then incubated in a primary antibody solution for 24 h at 4°C. Primary antibodies, dilutions, and validation information are included in Table 2. Sections were washed in PBS, incubated for 1 h in a biotinylated secondary antibody (Vector Laboratories), and incubated for 1 h in an avidin-biotin complex solution (1:200; Vector Laboratories). The chromogen nickel-enhanced diaminobenzidine (Sigma-Aldrich) was used to visualize immunoreactivity. Tissue was mounted onto slides, dehydrated, and cover-slipped. Negative control for non-specific binding was conducted on separate sections employing the above-mentioned procedures omitting the primary antibody.

### 2.10 Microscopic quantification and image analysis

Across experiments, BioQuant Nova Advanced Image Analysis software (R&M Biometric, Nashville, TN, USA) was used for image capture and quantification of immunohistochemistry. Representative images were captured using an Olympus BX50 microscope and Sony DXC390 video camera linked to a computer. For each measure, the microscope, camera, and software were background corrected and normalized to preset light levels to ensure fidelity of data acquisition. Microscopic quantification was conducted by individuals blinded to experimental treatment condition.

In the human basal forebrain, a modified stereological quantification method was used to quantify Aβ1-42+IR cells, as we previously reported that comparison of unbiased stereological methodology with our modified stereological approach yielded nearly identical values relative to control subjects (Crews et al., 2004). The outlined regions of interest were determined and data expressed as cells/mm^2^. Pixel density was used to assess Iba-1 and CD68 immunoreactivity in the human basal forebrain. Pixel density was rigorously thresholded to normalize pixel intensity (Vetreno and Crews, 2015) with the threshold for pixel density being determined from control subjects by calculating the average of the darkest and lightest values, and sections were imaged under identical conditions to avoid non-systematic variation (Beynon and Walker, 2012). The outlined regions of interest were determined and pixel density calculated by dividing the pixel count by the overall area (mm^2^).

In the mouse basal forebrain, microscopic quantification was conducted in the medial septum and vertical and horizontal limb of the diagonal band. Pixel density was used to assess Iba-1, CD68, RAGE, and pNFκBp65, and a modified unbiased stereological quantification method was used to assess ChAT and HMGB1 immunoreactivity as described above.

### 2.11 RNA extraction and reverse transcription PCR (RTPCR)

Total RNA was extracted from dissected mouse basal forebrain tissue samples by homogenization in TRI reagent (Sigma-Aldrich) following the single-step method of RNA isolation (Chomczynski and Sacchi, 2006). RNA quality and concentration were determined using a Qubit Fluorometer (Qubit™ 4 Fluorometer; Invitrogen, Carlsbad, CA, USA) in combination with a Qubit™ RNA Broad Range Assay kit (Invitrogen). Total RNA was reverse transcribed as previously described (Vetreno et al., 2021). RTPCR was run on a Bio-Rad CFX system (Bio-Rad Laboratories, Hercules, CA, USA) using SYBER Green PCR Master Mix (Life Technologies). RTPCR was run with an initial activation for 10 min at 95°C, followed by 40 cycles of denaturation (95°C, 15 s), annealing/extension (57–58°C, 1 min), and melt curve. The primer sequences are presented in Table 3. Differences in primer expression between groups are expressed as cycle time (Ct) values normalized with housekeeping genes and relative differences between groups calculated and expressed as the percent difference relative to CONs.

### 2.12 Statistical analysis

Statistical analysis was performed using SPSS (IBM; Chicago, IL, USA) and GraphPad Software (Prism 9; San Diego, CA, USA). For the post-mortem human analyses, two-tailed Student’s *t*-tests were used to assess demographics, RTPCR, and immunohistochemistry data unless otherwise reported. Levene’s Test for Equality of Variances was performed for each analysis. When reported in the Results, Welch’s *t*-tests were used to assess data with unequal variances. Two-tailed Pearson’s *r* were used for all correlative analyses. For the mouse analyses, repeated-measures ANOVAs were used to assess body weight and drinking behavior. Two-tailed Student’s *t*-tests and multivariate analysis of variance (MANOVA) with follow up Bonferroni corrections were used for the RTPCR data as reported in the Results. The immunohistochemical data was analyzed using 2 × 2 ANOVAs with appropriate *post-hoc* tests performed where appropriate as reported in the Results. All values are reported as mean ± SEM, and significance was defined as *p* < 0.05.

## 3 Results

### 3.1 Increased AD-associated neuropathology in the post-mortem human basal forebrain of individuals with AUD

**FIGURE 2:**
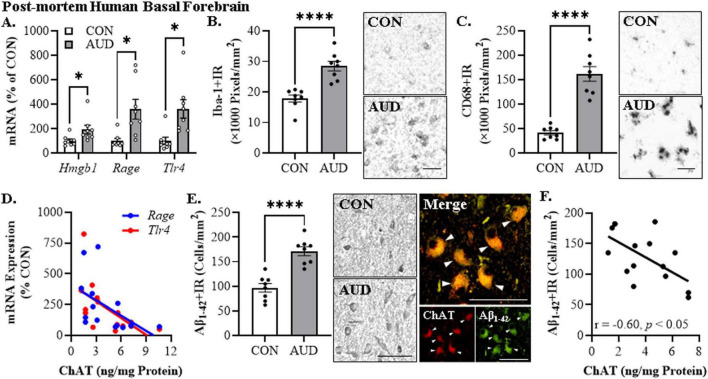
Alzheimer’s disease-associated neuropathology in the post-mortem human basal forebrain of individuals with alcohol use disorder (AUD). **(A)** Reverse transcription PCR (RTPCR) analysis revealed a 1.9-fold increase of *Hmgb1*, 3.6-fold increase of *Rage*, and a 3.6-fold increase of *Tlr4* mRNA in the post-mortem human basal forebrain of individuals with AUD, relative to age-matched moderate drinking CONs (*n* = 8/group, two-tailed *t*-tests). **(B)** Modified unbiased stereological assessment revealed a 1.6-fold increase of Iba-1+IR microglia in the post-mortem human basal forebrain of individuals with AUD, relative to age-matched moderate drinking CONs (*n* = 8/group, two-tailed *t*-test). Representative photomicrographs of Iba-1+IR in the basal forebrain of a CON and AUD sample. **(C)** Modified unbiased stereological assessment revealed a 3.9-fold increase of CD68+IR microglia in the post-mortem human basal forebrain of individuals with AUD, relative to age-matched moderate drinking CONs (*n* = 8/group, two-tailed *t*-test). Representative photomicrographs of CD68+IR in the basal forebrain of a CON and AUD sample. **(D)** Across individuals, protein expression levels of the acetylcholine-synthesizing enzyme choline acetyltransferase (ChAT; Vetreno et al., 2014) negatively correlated with mRNA expression of *Rage* (*r* = –0.52, *N* = 16) and *Tlr4* (*r* = –0.60, *N* = 16) in the post-mortem human basal forebrain (Pearson’s *r* correlation). **(E)** Modified unbiased stereological assessment revealed a 1.8-fold increase of Aβ_1–42_+IR in the post-mortem human basal forebrain of individuals with AUD, relative to age-matched moderate drinking CONs (*n* = 8/group, two-tailed *t*-test). Representative photomicrographs of Aβ_1–42_+IR in the basal forebrain of a CON and AUD sample. Double immunofluorescent analysis revealed an approximate 1.3-fold increase of Aβ_1–42_+IR co-expression with ChAT in the basal forebrain of individuals with AUD relative to age-matched moderate drinking CONs. Photomicrographs of ChAT (RED) colocalization with Aβ_1–42_ (RED) in the post-mortem human basal forebrain of an individual with AUD. White arrows indicate ChAT+IR cells that colocalize with Aβ_1–42_ (YELLOW). **(F)** Across individuals, protein expression of ChAT negatively correlates with immunohistochemical expression of Aβ_1–42_ (*r* = –0.60, *N* = 15) in the post-mortem human basal forebrain (Pearson’s *r* correlation). Scale bar = 30 μm. RTPCR run in duplicate. Data presented as mean ± SEM. *p < 0.05, ****p < 0.0001.

To determine if a history of AUD increases AD-associated neuropathology, we first assessed proinflammatory signaling molecules in the post-mortem human basal forebrain of individuals with AUD (age of death = 49 ± 2 years) relative to age-matched moderate drinking CONs (age of death = 48 ± 4 years). We observed an approximate 1.9-fold, 3.6-fold, and 3.6-fold increase of *Hmgb1* [*t*(9.3) = 2.4, *p* = 0.042, Welch’s *t*-test], *Rage* [*t*(7.9) = 3.1, *p* = 0.015, Welch’s *t*-test], and *Tlr4* [*t*(9.2) = 3.2, *p* = 0.010, Welch’s *t*-test] mRNA, respectively, in the basal forebrain of individuals with AUD relative to age-matched CONs (see Figure 2A). Microglial activation is thought to contribute to the proinflammatory signaling observed in AD as well as the progression of AD-associated neuropathology (Regen et al., 2017; Hansen et al., 2018; Streit et al., 2018; Leng and Edison, 2021). Immunohistochemical assessment of the pan-microglial marker Iba-1 revealed an approximate 1.6-fold increase in the AUD basal forebrain [*t*(14) = 5.5, *p* = 0.0001, Student’s *t*-test] relative to moderate drinking CONs (see Figure 2B). Similarly, immunohistological assessment of CD68+IR microglia revealed an amoeboid change in microglial morphology in the AUD basal forebrain consistent with microglial activation. Quantification of CD68+IR revealed an approximate 3.9-fold increase in the AUD basal forebrain [*t*(8.2) = 7.8, *p* = 0.0001, Welch’s *t*-test] relative to moderate drinking CONs (see Figure 2C). We previously reported a reduction of the acetylcholine-synthesizing enzyme choline acetyltransferase (ChAT), which is a marker of cholinergic neurons, in the post-mortem basal forebrain of individuals with AUD (Vetreno et al., 2014), and degeneration of cholinergic neurons within this region is a hallmark feature of AD (Nagai et al., 1983; McGeer et al., 1984; Lehericy et al., 1993). Our prior study included several patients overlapping with the current study, allowing correlational assessment of ChAT protein levels with proinflammatory signaling molecules. Expression of ChAT protein in the post-mortem human basal forebrain negatively correlated with *Rage* (*r* = −0.52, *p* = 0.048, Pearson’s *r*) and *Tlr4* (*r* = −0.60, *p* = 0.017, Pearson’s *r*) mRNA, suggesting that neuroinflammation may contribute to the loss of cholinergic neurons (Crews and Vetreno, 2022; Crews et al., 2023; see Figure 2D). Thus, these data suggest that neuroinflammatory HMGB1-TLR4/RAGE signaling and microglial activation is induced in the post-mortem basal forebrain of individuals with a history of heavy alcohol use which may contribute to reductions of cholinergic neurons.

We next assessed whether a history of heavy alcohol consumption is associated with amyloid (Aβ) pathology in the post-mortem human basal forebrain. Using an antibody against the Aβ_1–42_ cleaved peptide, which is considered the neurotoxic form of Aβ (Billings et al., 2005), we discovered an approximate 1.8-fold increase of Aβ_1–42_+IR cells in the basal forebrain of individuals with AUD [*t*(14) = 5.9, *p* = 0.0001, Student’s *t*-test] relative to moderate drinking CONs (see Figure 2E). Given the neuron-like immunohistological profile observed, we next assessed co-expression of Aβ_1–42_ with ChAT+IR cholinergic neurons. Double immunofluorescent analysis revealed an approximate 1.3-fold increase of Aβ_1–42_+IR co-expression with ChAT in the basal forebrain of individuals with AUD [*t*(13) = 2.7, *p* = 0.019, Student’s *t*-test] relative to age-matched moderate drinking CONs. Further, expression of Aβ_1–42_+IR negatively correlated with ChAT protein expression in the basal forebrain (*r* = −0.60, *p* = 0.018, Pearson’s *r*) (see Figure 2F). These data suggest that the pattern of heavy alcohol consumption that characterizes AUD increases intraneuronal Aβ_1–42_ in ChAT+IR neurons that may also contribute to loss of cholinergic neurons.

Since an earlier age of drinking onset is associated with increased risk for later development of AUD (Grant and Dawson, 1997; Grant et al., 2001), we next correlated age of drinking onset with AD-associated neuropathology. Age of drinking onset, which ranged in AUD individuals from 14 to 20 years of age, was negatively correlated with *Rage* (*r* = −0.61, *p* = 0.012, Pearson’s *r*), *Tlr4* (*r* = −0.53, *p* = 0.035, Pearson’s *r*), Iba-1+IR (*r* = −0.63, *p* = 0.005, Pearson’s *r*), CD68+IR (*r* = −0.88, *p* = 0.00003, Pearson’s *r*), and Aβ_1–42_+IR (*r* = −0.80, *p* = 0.0002, Pearson’s *r*), and positively correlated with expression of ChAT (*r* = 0.49, *p* = 0.032, Pearson’s *r*), suggesting that a younger age of drinking onset is associated with increased AD-associated neuropathology in the adult basal forebrain. In this cohort, AUD individuals consumed between 613 kg and 2,452 kg of alcohol across their lifetime, providing a broad range of consumption levels that parallels progressive increases of AD-associated markers. Expression of Iba-1+IR (*r* = 0.56, *p* = 0.025, Pearson’s *r*), CD68+IR (*r* = 0.61, *p* = 0.036, Pearson’s *r*), and Aβ_1–42_+IR (*r* = 0.70, *p* = 0.011, Pearson’s *r*) positively correlated with lifetime alcohol consumption. These findings are consistent with early onset, heavy alcohol use contributing to increases in AD-associated neuropathology, including induction of proinflammatory genes, alterations in microglial marker expression consistent with a shift to a proinflammatory state, and increased Aβ_1–42_ in cholinergic neurons of the basal forebrain in association with loss of cholinergic neuron markers. Taken together, these data suggest that an adolescent age of drinking onset may contribute to development of AD-associated neuropathology.

### 3.2 Adolescent binge ethanol exposure accelerates AD-associated neuropathology in the adult female basal forebrain of 5xFAD mice

**FIGURE 3:**
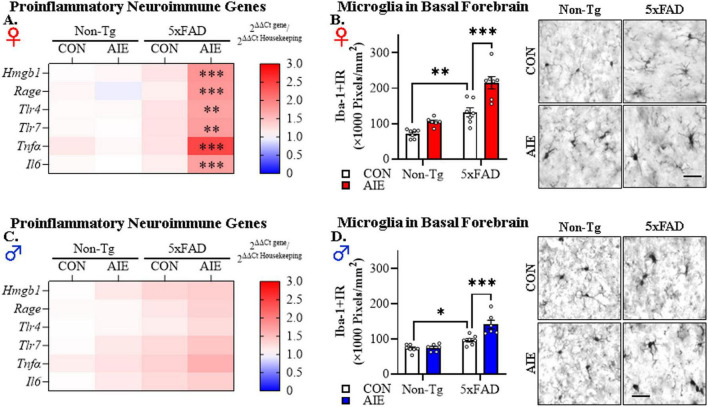
Adolescent intermittent ethanol (AIE) treatment accelerates Alzheimer’s disease-associated neuroinflammation in the adult basal forebrain of female 5xFAD mice. **(A)** Heat map of reverse transcription PCR (RTPCR) analyses reveals AIE-induced increases of the proinflammatory genes *Hmgb1* (1.7-fold), *Rage* (1.8-fold), *Tlr4* (1.6-fold), *Tlr7* (1.6-fold), *Tnf*α (2.5-fold), and *Il6* (1.7-fold) in the adult female basal forebrain of AIE-treated 5xFAD mice, relative to CON-treated female 5xFAD mice (*n* = 6–7/group, MANOVA with Bonferroni correction). **(B)** Modified unbiased stereological assessment revealed that constitutive immunohistochemical expression of the microglial marker Iba-1 increased approximately 1.9-fold in CON-treated female 5xFAD mice relative to Non-Tg CONs whereas AIE treatment increased Iba-1+IR 1.6-fold in female AIE-treated 5xFAD mice relative to CON-treated 5xFAD mice (*n* = 6–7/group, 2 × 2 ANOVA with Bonferroni correction). Representative photomicrographs of Iba-1+IR in the adult female basal forebrain of a CON- and AIE-treated Non-Tg and 5xFAD mouse. **(C)** Heat map of RTPCR analyses reveals that mRNA expression of proinflammatory genes are unchanged in the adult male basal forebrain (*n* = 7/group, MANOVA with Bonferroni correction). **(D)** Modified unbiased stereological assessment revealed that AIE treatment caused a modest 1.4-fold increase of Iba-1+IR in the adult basal forebrain of AIE-treated 5xFAD mice relative to CON-treated 5xFAD mice (*n* = 7/group, 2 × 2 ANOVA with Bonferroni correction). Representative photomicrographs of Iba-1+IR in the adult male basal forebrain of a CON- and AIE-treated Non-Tg and 5xFAD mouse. Scale bar = 30 μm. RTPCR run in duplicate. Data presented as mean ± SEM. *p < 0.05, **p < 0.01, ***p < 0.001.

To investigate whether AIE treatment affects AD-associated neuroinflammation in adulthood, we first assessed mRNA expression of proinflammatory neuroimmune genes and microglial marker expression in the basal forebrain of adult (i.e., P100) female and male Non-Tg and 5xFAD mice following AIE treatment. We model human adolescent binge drinking behavior using an intermittent ethanol administration schedule consistent with known patterns of heavy weekend binge drinking, but not daily drinking associated with AUD in adulthood. Assessment of proinflammatory neuroimmune genes revealed a trend toward a significant interaction between Sex, Genotype, and Treatment on the combined dependent variables [*F*(6, 40) = 2.18, *p* = 0.066; Wilks’ Λ = 0.754, MANOVA]. Follow-up pairwise comparisons revealed that AIE treatment did not affect expression of proinflammatory neuroimmune genes in the adult Non-Tg female or male basal forebrain. In contrast, in adult 5xFAD female mice, AIE treatment increased mRNA expression of *Hmgb1* (1.7-fold; *p* = 0.0003, Bonferroni Correction), *Rage* (1.8-fold; *p* = 0.0005, Bonferroni Correction), *Tlr4* (1.6-fold; *p* = 0.004, Bonferroni Correction), *Tlr7* (1.6-fold; *p* = 0.005, Bonferroni Correction), *Tnf*α (2.5-fold; *p* = 0.0003, Bonferroni Correction), and *Il6* (1.7-fold; *p* = 0.0009, Bonferroni Correction) relative to CON-treated female 5xFAD mice (see Figure 3A). In 5xFAD male mice, AIE treatment did not affect expression of proinflammatory neuroimmune genes (see Figure 3C). Immunohistological assessment of microglial Iba-1+IR expression revealed increased Iba-1+IR in the female basal forebrain relative to males {significant main effect of Sex [*F*(1, 45) = 25.3, *p* = 0.000008, 2 × 2 × 2 ANOVA]}. In the females, follow-up pairwise comparisons of the significant interaction of Treatment × Genotype [*F*(1, 23) = 4.6, *p* = 0.042] revealed a 1.9-fold increase of Iba-1+IR in CON-treated 5xFAD mice (*p* = 0.001, Bonferroni Correction), relative to CON-treated Non-Tg mice. Interestingly, AIE treatment caused a 1.6-fold increase of Iba-1+IR in 5xFAD female mice, relative to 5xFAD CONs (*p* = 0.00003, Bonferroni Correction; see Figure 3B). In the males, follow-up pairwise comparisons of the significant interaction of Treatment × Genotype [*F*(1, 22) = 9.4, *p* = 0.006] revealed a 1.3-fold increase of Iba-1+IR in CON-treated 5xFAD mice (*p* = 0.020, Bonferroni Correction), relative to CON-treated Non-Tg mice. Prior AIE treatment increased Iba-1+IR by 1.4-fold in the adult basal forebrain of male AIE-treated 5xFAD mice relative to CON-treated 5xFAD mice (*p* = 0.00002, Bonferroni Correction; see Figure 3D). These data suggest that adolescent binge ethanol exposure accelerates AD-associated proinflammatory neuroimmune gene induction in the adult female basal forebrain and causes microglial morphological changes across sexes, albeit to a greater extent in the females, consistent with acceleration of proinflammatory neuroimmune activation in the adult female basal forebrain of 5xFAD mice.

**FIGURE 4:**
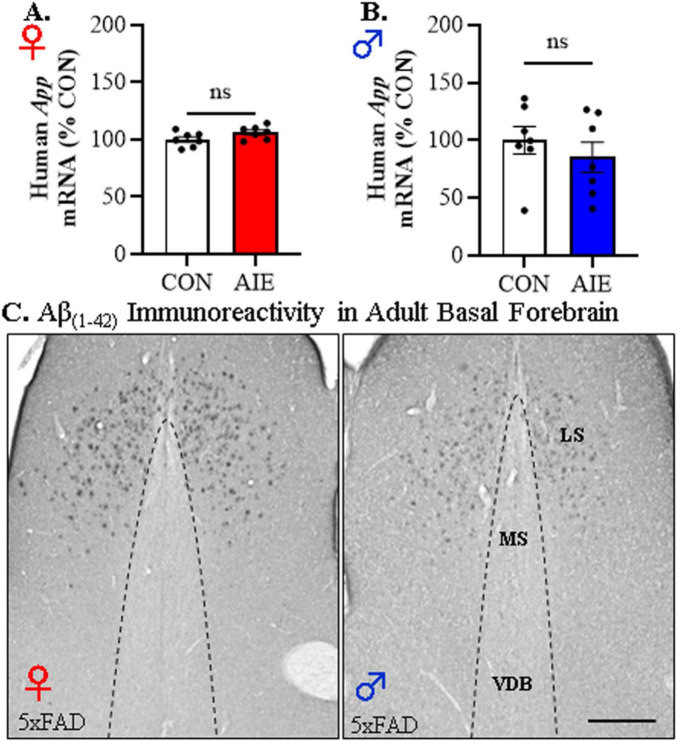
Adolescent intermittent ethanol (AIE) does not affect human *App* transgene expression or Aβ_1–42_ plaque deposition in the adult basal forebrain of 5xFAD mice. Reverse transcription PCR (RTPCR) analysis of the human amyloid precursor protein (*App*) revealed that AIE treatment did not alter *App* gene expression in the adult **(A)** female (*n* = 7/group, two-tailed *t*-test) or **(B)** male (*n* = 7/group, two-tailed *t*-test) basal forebrain relative to age-matched CON-treated 5xFAD mice. **(C)** Representative photomicrographs of Aβ_1–42_+IR in the basal forebrain of an adult AIE-treated male and female 5xFAD mouse. Immunohistochemical analysis revealed an absence of Aβ_1–42_+IR cells or plaques in the medial septum (MS) or vertical limb of the diagonal band (VDB) of the basal forebrain whereas deposition of Aβ_1–42_+IR plaques was observed in the lateral septum (LS). Scale bar = 100 μm. RTPCR run in duplicate. Data presented as mean ± SEM.

We next assessed amyloid pathology to determine if AIE treatment promotes early accumulation of Aβ_1–42_ in the adult basal forebrain. Expression of the human *App* transgene in the basal forebrain was unchanged by AIE treatment in adult female [*t*(12) = 1.9, *p* = 0.07, Student’s *t*-test; see Figure 4A] or male [*t*(12) = 0.83, *p* = 0.82, Student’s *t*-test; see Figure 4B] 5xFAD mice. While immunohistological assessment of Aβ_1–42_ revealed plaque deposits in the lateral septum, we observed an absence of positive intraneuronal Aβ_1–42_ staining or evidence of plaque deposition in the adult basal forebrain of male and female 5xFAD mice (see Figure 4C). These data suggest that Aβ_1–42_ plaque deposition may not occur in regions of the basal forebrain that cholinergic neurons populate.

**FIGURE 5:**
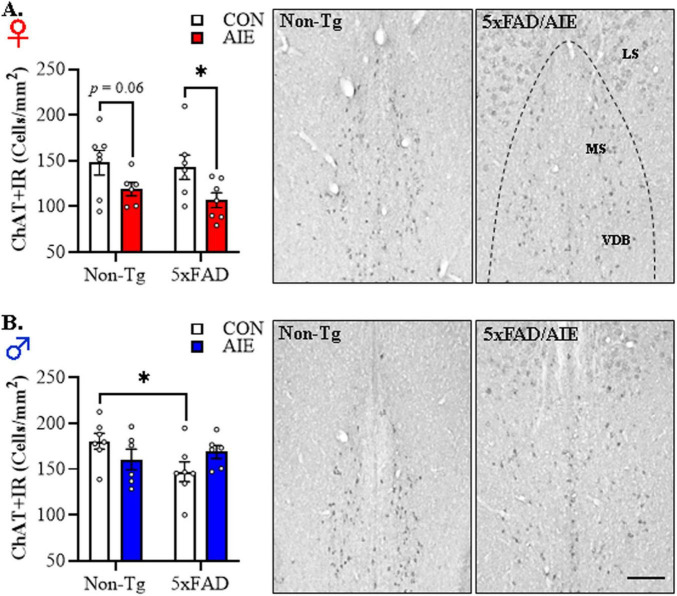
Adolescent intermittent ethanol (AIE) accelerates Alzheimer’s disease-associated reductions of cholinergic neurons in the adult basal forebrain of female 5xFAD mice. **(A)** Modified unbiased stereological assessment revealed that AIE treatment decreased choline acetyltransferase (ChAT)+IR cholinergic neurons in the adult 5xFAD female basal forebrain relative to 5xFAD CONs (*n* = 6–7/group, 2 × 2 ANOVA with Bonferroni correction). Representative photomicrographs of ChAT+IR cholinergic neurons in the adult female basal forebrain of a CON-treated Non-Tg and AIE-treated 5xFAD mouse. **(B)** Modified unbiased stereological assessment revealed that ChAT+ cholinergic neuron populations were reduced in adult 5xFAD CON male whereas AIE treatment did not affect ChAT expression in males (*n* = 6–7/group, 2 × 2 ANOVA with Bonferroni correction). Representative photomicrographs of ChAT+IR cholinergic neurons in the adult male basal forebrain of a CON-treated Non-Tg and AIE-treated 5xFAD mouse. MS, medial septum; VDB, vertical limb of the diagonal band; HDB, horizontal limb of the diagonal band. Scale bar = 50 μm. Data presented as mean ± SEM. *p < 0.05.

In our preclinical rat AIE model, we previously reported a loss of basal forebrain cholinergic neurons immediately following the conclusion of AIE treatment that persists into adulthood in the absence of continued ethanol exposure (Vetreno et al., 2014; Crews et al., 2021). Basal forebrain cholinergic system degeneration is a hallmark feature of human AD (Lehericy et al., 1993; Beach et al., 2000; Stepanichev et al., 2017; Hampel et al., 2018), but the role of adolescent binge alcohol exposure in AD-associated cholinergic degeneration is unknown. Thus, we next assessed whether AIE treatment affects cholinergic neuron population in the adult basal forebrain of female and male 5xFAD mice relative to age-matched Non-Tg littermates. Across basal forebrain tissue samples, assessment of ChAT+IR neurons revealed darkly stained cell bodies and processes. Immunohistological assessment of ChAT+IR revealed a significant main effect of Sex [*F*(1, 45) = 22.4, *p* = 0.00002, 2 × 2 × 2 ANOVA] and a trend toward a significant interaction between Sex, Genotype, and Treatment [*F*(1, 45) = 2.7, *p* = 0.10, 2 × 2 × 2 ANOVA; see Figures 5A, B]. Follow-up pairwise comparisons revealed a tread toward an AIE-induced reduction of ChAT+IR in the female Non-Tg basal forebrain (*p* = 0.060, Bonferroni Correction) relative to Non-Tg CONs, and a significant AIE-induced 25% reduction of ChAT+IR neurons in the female 5xFAD basal forebrain (*p* = 0.015, Bonferroni Correction) relative to CON-treated 5xFAD mice (see Figure 5A). In males, we observed a reduction of ChAT+IR cholinergic neurons in CON-treated 5xFAD mice relative to Non-Tg CONs, whereas prior AIE treatment did not affect ChAT expression in males (see Figure 5B). During our assessment of ChAT+IR cholinergic neurons, we observed plaque-like ChAT+ staining in the lateral septum similar to the expression pattern of Aβ_1–42_. Consequent immunohistochemical staining of ChAT with a ChAT-blocking peptide blocked expression of ChAT+IR neurons, but did not block formation of the observed ChAT-like plaques. Thus, the plaque-like ChAT+ staining is likely a consequence of non-specific staining likely due to the “sticky” nature of amyloid plaques (see Supplementary Figure 2). These data suggest that adolescent binge ethanol exposure in females, but not males, causes a lasting reduction of basal forebrain cholinergic neurons that may provide an early marker of AD progression (Grothe et al., 2012; Schmitz et al., 2016). Taken together, early life insults, such as adolescent binge drinking, may represent an etiological factor contributing to the risk for later AD development through initiation of persistent neuroinflammation and microglial activation as well as lasting reductions of basal forebrain cholinergic neurons. Further, our observation of more robust AD-associated neuropathology in the female basal forebrain is consistent with the higher incidence of AD diagnosis in human females (Rocca et al., 1986; Beam et al., 2018).

### 3.3 Administration of the selective HMGB1 inhibitor glycyrrhizic acid following the conclusion of AIE reverses acceleration of AD-associated neuropathology in the adult basal forebrain of 5xFAD female mice

**FIGURE 6:**
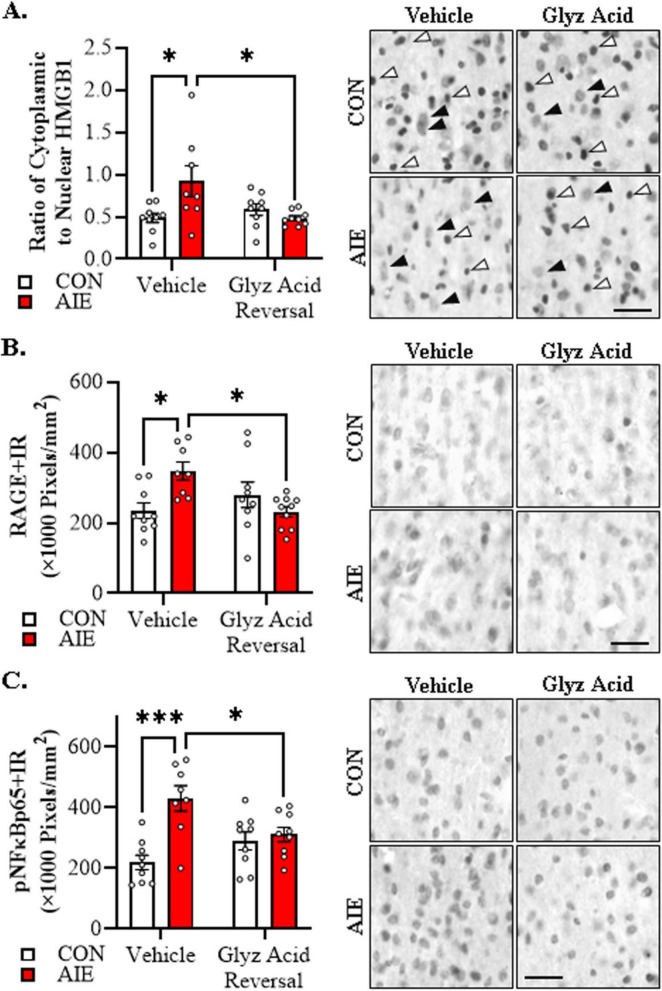
Glycyrrhizic acid treatment reverses the adolescent intermittent ethanol (AIE)-induced acceleration of Alzheimer’s disease-associated neuroinflammation in the adult basal forebrain of female 5xFAD mice. **(A)** Modified unbiased stereological assessment revealed a 1.9-fold increase of cytoplasmic HMGB1+IR in basal forebrain of AIE-treated female 5xFAD mice relative to CONs. While glycyrrhizic acid treatment alone did not affect cytoplasmic HMGB1 expression in CON-treated 5xFAD mice, it reversed the AIE-induced basal forebrain acceleration of HMGB1 in the cytoplasm of 5xFAD mice (*n* = 8–9/group, 2 × 2 ANOVA with Tukey’s HSD). Representative photomicrographs of HMGB1+IR in the adult female basal forebrain of a vehicle- and glycyrrhizic acid-treated CON and AIE 5xFAD mouse. White arrows indicate HMGB1+IR nuclear staining and black arrows indicate HMGB1+IR cytoplasmic staining. **(B)** Modified unbiased stereological assessment revealed a 1.5-fold increase of RAGE+IR in the adult basal forebrain of AIE-treated female 5xFAD mice relative to CONs. Glycyrrhizic acid treatment alone did not affect RAGE expression in CON-treated 5xFAD mice, but did reverse the acceleration of RAGE expression in AIE-treated 5xFAD mice (*n* = 8–10/group, 2 × 2 ANOVA with Tukey’s HSD). Representative photomicrographs of RAGE+IR in the adult female basal forebrain of a vehicle- and glycyrrhizic acid-treated CON and AIE 5xFAD mouse. **(C)** Modified unbiased stereological assessment revealed a 2.0-fold increase of activated pNFκB p65+IR in the basal forebrain of AIE-treated female 5xFAD mice relative to CONs. While glycyrrhizic acid treatment did not affect expression of pNFκB p65 in CON-treated 5xFAD mice, it did reverse the AIE-induced acceleration of pNFκB p65+IR in 5xFAD mice (*n* = 8–9/group, 2 × 2 ANOVA with Tukey’s HSD). Representative photomicrographs of pNFκB p65+IR in the adult female basal forebrain of a vehicle- and glycyrrhizic acid-treated CON and AIE 5xFAD mouse. Scale bar = 25 μm. Data presented as mean ± SEM. *p < 0.05, ***p < 0.001.

We next assessed whether administration of glycyrrhizic acid, a small molecule selective inhibitor of HMGB1, reverses the AIE-induced acceleration of AD-associated neuropathology. In the current study, we focused on female 5xFAD mice given our findings that AIE accelerates AD-associated neuropathology in female mice. Immunohistological assessment of HMGB1 revealed that AIE treatment caused a 1.9-fold increase of cytoplasmic HMGB1+IR in female 5xFAD mice relative to CONs (Tukey’s HSD: *p* = 0.018). Importantly, post-AIE administration of glycyrrhizic acid reversed the AIE-induced increase of cytoplasmic HMGB1 relative to vehicle-treated AIE mice (Tukey’s HSD: *p* = 0.016; see Figure 6A). Assessment of RAGE expression revealed that AIE caused a 1.5-fold increase of RAGE+IR in the adult female 5xFAD basal forebrain relative to CONs (Tukey’s HSD: *p* = 0.023), an effect that was reversed by post-AIE glycyrrhizic acid administration (Tukey’s HSD: *p* = 0.015; see Figure 6B). HMGB1 activation of TLR4 and RAGE leads to downstream activation of NFκB (Chavakis et al., 2004; Tobon-Velasco et al., 2014; Liu et al., 2017; Aucott et al., 2018). NFκB is a nuclear transcription factor known to induce multiple neuroimmune genes (Pahl, 1999), and activated pNFκB p65 provides insight into proinflammatory neuroimmune signaling in brain (Vetreno et al., 2017; Vetreno and Crews, 2018; Vetreno et al., 2018). We report a significant AIE-induced 2.0-fold increase of pNFκB p65+IR cells in the adult basal forebrain relative to CON subjects (Tukey’s HSD: *p* = 0.0001). While glycyrrhizic acid treatment alone did not affect pNF-κB p65+IR, it reversed the AIE-induced increase of pNFκB p65+IR relative to vehicle-treated AIE subjects (Tukey’s HSD: *p* = 0.043; see Figure 6C). Thus, these data suggest that post-AIE treatment with the HMGB1 inhibitor glycyrrhizic acid exerts anti-inflammatory effects through reversal of AIE-induced increases of HMGB1-RAGE- pNFκB p65 signaling.

**FIGURE 7:**
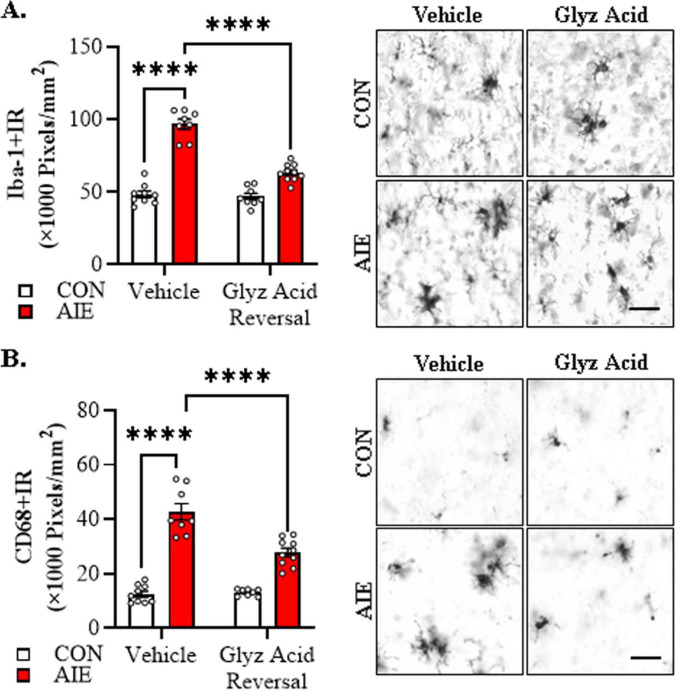
Post-adolescent intermittent ethanol (AIE) treatment with glycyrrhizic acid reverses acceleration of Alzheimer’s disease-associated microglial activation in the adult basal forebrain of female 5xFAD mice. **(A)** Modified unbiased stereological assessment revealed a 2.0-fold increase of Iba-1+IR in the adult basal forebrain of AIE-treated 5xFAD mice relative to CONs. While glycyrrhizic acid treatment alone did not affect Iba-1 expression in CONs, it reversed the AIE-induced acceleration of Iba-1+IR relative to vehicle-treated AIE mice (*n* = 8–10/group, 2 × 2 ANOVA with Tukey’s HSD). Representative photomicrographs of Iba-1+IR in the adult female basal forebrain of a vehicle- and glycyrrhizic acid-treated CON and AIE 5xFAD mouse. **(B)** Modified unbiased stereological assessment revealed a 4.0-fold increase of CD68+IR in the adult basal forebrain of AIE-treated female 5xFAD mice relative to CONs. While glycyrrhizic acid treatment alone did not affect CD68 expression in CONs, it reversed the AIE-induced increase of CD68+IR relative to vehicle-treated AIE mice (*n* = 8–10/group, 2 × 2 ANOVA with Tukey’s HSD). Representative photomicrographs of CD68+IR in the adult female basal forebrain of a vehicle- and glycyrrhizic acid-treated CON and AIE 5xFAD mouse. Scale bar = 30 μm. Data presented as mean ± SEM. ****p < 0.0001.

We next sought to determine if post-AIE administration of glycyrrhizic acid reverses alterations in microglia morphology in the adult basal forebrain following AIE treatment. We found that AIE treatment caused a 2.0-fold increase in Iba-1+IR in the adult basal forebrain relative to CON subjects (Tukey’s HSD: *p* = 0.0001). While glycyrrhizic acid treatment alone did not affect Iba-1+IR, it reversed the AIE-induced increase of Iba-1+IR relative to vehicle-treated AIE subjects (Tukey’s HSD: *p* = 0.0001; see Figure 7A). Immunohistological assessment of CD68+IR microglia revealed amoeboid morphological changes of microglia in the AIE-treated adult basal forebrain, consistent with microglial activation. Quantification of CD68+IR microglia revealed a 4.0-fold increase in CD68+IR in the adult basal forebrain relative to CON subjects (Tukey’s HSD: *p* = 0.0001). Glycyrrhizic acid treatment alone did not affect CD68+IR in CONs but did partially reverse the AIE-induced increase of CD68+IR relative to vehicle-treated AIE subjects (Tukey’s HSD: *p* = 0.0001; see Figure 7B). Thus, treatment with the HMGB1 inhibitor glycyrrhizic acid reverses AIE-induced microglial morphology changes, consistent with reversal of AIE-induced acceleration of microglial activation in the adult basal forebrain.

**FIGURE 8:**
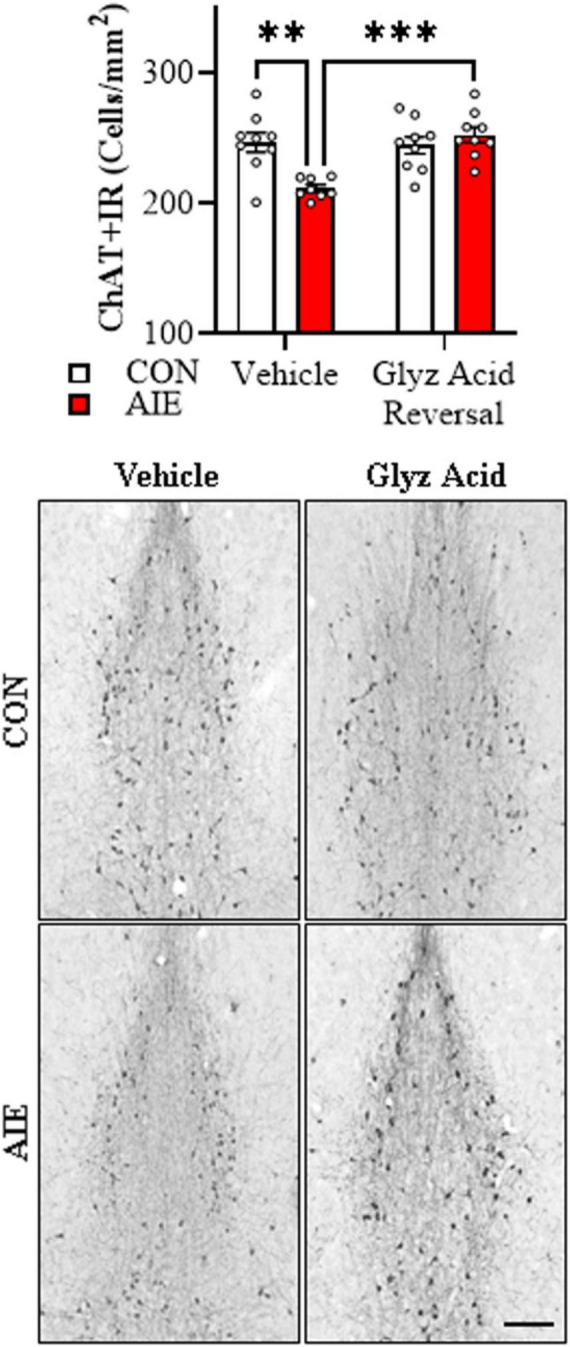
Glycyrrhizic acid treatment reverses the adolescent intermittent ethanol (AIE)-induced acceleration of Alzheimer’s disease-associated basal forebrain cholinergic neuron loss in the adult female 5xFAD mice. Modified unbiased stereological assessment revealed a 15% reduction of ChAT+IR cholinergic neurons in the adult basal forebrain of AIE-treated 5xFAD mice relative to vehicle-treated AIE subjects. While glycyrrhizic acid treatment alone did not affect ChAT expression in CONs, it reversed the AIE-induced acceleration of ChAT+IR cholinergic neuron loss in 5xFAD mice (*n* = 8–10/group, 2 × 2 ANOVA with Tukey’s HSD). Representative photomicrographs of ChAT+IR in the adult female basal forebrain of a vehicle- and glycyrrhizic acid-treated CON and AIE 5xFAD mouse. Scale bar = 50 μm. Data presented as mean ± SEM. **p < 0.01, ***p < 0.001.

Given our findings of reduced expression of cholinergic neurons in the post-mortem human AUD basal forebrain and adult basal forebrain of female AIE-treated mice, we next assessed whether post-AIE treatment with glycyrrhizic acid reverses the AIE-induced loss of ChAT+ cholinergic neurons in adult 5xFAD mice. Consistent with our previous study, AIE treatment caused a 15% (± 1.1%) reduction of ChAT+IR cholinergic neurons in the adult basal forebrain, relative to vehicle-treated CON subjects (Tukey’s HSD: *p* = 0.002). While glycyrrhizic acid treatment did not affect ChAT+IR neuron populations in CONs, it reversed the AIE-induced acceleration of AD-associated ChAT+ basal forebrain cholinergic neuron loss (Tukey’s HSD: *p* = 0.0003; see Figure 8). Taken together, post-AIE treatment with the selective HMGB1 inhibitor glycyrrhizic acid reverses AIE-induced acceleration of AD-associated neuroinflammation, microglial activation, and loss of cholinergic neurons in the adult basal forebrain.

## 4 Discussion

Emerging epidemiological studies implicate heavy alcohol use and AUD as a risk factor for later development of dementia and AD-associated neuropathology (Fratiglioni et al., 1993; Mukamal et al., 2003; Langballe et al., 2015; Schwarzinger et al., 2018). While adolescent alcohol abuse is associated with a host of deleterious outcomes, including increased risk for development of an AUD, the contribution of adolescent binge drinking to later development of AD-associated basal forebrain neuropathology is poorly understood. Degeneration of the basal forebrain cholinergic system is a hallmark of AD and occurs early in disease progression (Lehericy et al., 1993; Beach et al., 2000; Grothe et al., 2012; Schmitz et al., 2016; Stepanichev et al., 2017; Hampel et al., 2018). Our laboratory and others report that preclinical AIE treatment persistently reduces basal forebrain cholinergic neuron populations well into adulthood in the absence of continued alcohol exposure (Coleman et al., 2011; Ehlers et al., 2011; Vetreno et al., 2014; Swartzwelder et al., 2015; Fernandez and Savage, 2017; Vetreno and Crews, 2018). In the current study, we investigated the effect of adolescent onset of alcohol binge drinking and exposure on development of AD-associated basal forebrain neuropathology in post-mortem adult human AUD tissue samples and in the 5xFAD mouse model of AD. In post-mortem human basal forebrain from individuals with AUD and an adolescent age of drinking onset, we observed increased proinflammatory gene induction (i.e., *Hmgb1*, *Rage*, and *Tlr4*) and expression of microglial Iba-1 and CD68 immunoreactivity. Loss of basal forebrain cholinergic neuron markers (i.e., ChAT) in the AUD basal forebrain (Vetreno et al., 2014) is accompanied by increased Aβ_1–42_ immunoreactive cells as well as colocalization of Aβ_1–42_ with ChAT, consistent with increased intraneuronal accumulation of Aβ_1–42_ in cholinergic neurons. Intraneuronal accumulation of Aβ_1–42_ in the basal forebrain is posited to be an early event in the progression of AD (Arendt et al., 1988; Billings et al., 2005; LaFerla et al., 2007; Gouras et al., 2010; Baker-Nigh et al., 2015; Takahashi et al., 2017). Expression levels of proinflammatory genes, microglial markers, and Aβ_1–42_ in the post-mortem basal forebrain negatively correlated with expression of ChAT as well as age of drinking onset, suggesting that heavy alcohol use initiated at an early age (i.e., adolescence) may contribute to AD-associated neuropathology later in life. While these human data suggest that adolescent binge drinking may contribute to the onset of AD-associated neuropathology, lifetime alcohol consumption may also represent a mediating factor. Thus, direct etiological contributions of early life insults (i.e., binge drinking) to disease onset and progression remain to be fully elucidated. Thus, employing the preclinical 5xFAD transgenic mouse model, we discovered that AIE treatment accelerates induction of HMGB1 proinflammatory neuroimmune genes and microglial marker expression in the adult basal forebrain that parallels reductions of ChAT+ basal forebrain cholinergic neuron. Although we did not observe either Aβ_1–42_ intraneuronal accumulation or plaque deposition in the adult 5xFAD basal forebrain proper, this finding is consistent with human data reporting an absence of dense-core Aβ plaques in the post-mortem AD basal forebrain (Wu et al., 2005) and the transient nature of intraneuronal Aβ_1–42_ accumulation in 5xFAD mice (Eimer and Vassar, 2013). Proinflammatory neuroimmune signaling induced by AIE appears to contribute to the observed acceleration of AD-associated neuropathology as post-AIE treatment with the selective HMBG1 inhibitor glycyrrhizic acid reverses AIE-induced proinflammatory signaling, microglial morphological changes, and reductions of basal forebrain cholinergic neurons in the adult basal forebrain of 5xFAD female mice. Taken together, these human and rodent findings suggest that adolescent binge drinking induction of HMBG1 proinflammatory signaling may represent a critical etiological factor contributing to the onset and progression of AD-associated basal forebrain neuropathology.

In the current investigation, the AIE-induced acceleration of AD-associated neuropathology was particularly evident in female AIE-treated 5xFAD mice and lacking in males, consistent with sex representing a crucial biological variable contributing to vulnerability to AD-associated disease onset and progression. Indeed, over 60% of documented AD cases occur in females, and sex is a documented risk factor for AD development (Riedel et al., 2016). Although the underlying mechanism contributing to increased risk for AD development in females is unknown, several factors have been postulated, including longer lifespan of women, menopausal transitions, and mitochondrial toxicity of amyloid plaques (Riedel et al., 2016; Scheyer et al., 2018; Vina and Lloret, 2010). It is also notable that females appear to be more susceptible to alcohol-induced neurodegeneration and cognitive deficits (Hommer, 2003), further emphasizing the female vulnerability to accelerated AD pathology due to alcohol. Interestingly, accumulating evidence suggests there may be sex differences in efficacy of cholinesterase inhibitor treatment with some studies reporting that cholinesterase inhibitors may delay AD progression during the prodromal stages more efficaciously in women than men (Ferretti et al., 2018). This potential sex difference may be due to heightened vulnerability of the female basal forebrain cholinergic system, which is consistent with the preclinical 5xFAD findings in the current study.

Adolescence is a conserved neurodevelopmental period characterized by functional maturation of glia (e.g., microglia) and the innate immune system that parallels refinement of brain structures, neurotransmitter systems, and neurocircuitry. Microglia in the developing adolescent brain exhibit an activation-like morphology (Schwarz et al., 2011; Schwarz et al., 2012) and undergo brain regional maturation that parallels neural maturation (Scheffel et al., 2012; Brenhouse and Schwarz, 2016; Hanamsagar et al., 2018) which may affect their responsivity to an immune challenge (e.g., alcohol) during adolescence. Microglia express the entire repertoire of TLRs whereas expression of TLRs by other cells of the brain, including neurons, are differentially expressed across neurodevelopment into adulthood (Olson and Miller, 2004; Okun et al., 2011). Similarly, expression levels of TLR4 and RAGE decrease in the rat cortex from adolescence into adulthood whereas HMGB1 levels increase during this period (Vetreno and Crews, 2012; Vetreno et al., 2013), consistent with adolescent maturation of the innate immune system. Adolescent development of microglia and the innate immune system coincides with maturational refinement and consolidation of basal forebrain cholinergic neurons, their projections, and cholinergic receptors (Matthews et al., 1974; Nadler et al., 1974; Zahalka et al., 1993). The increased plasticity associated with cholinergic neuron development, coupled with maturation of the innate immune system, increases vulnerability of the adolescent brain to shifts in developmental trajectory in response to insult. Thus, stimuli that negatively impact immature adolescent microglia, such as adolescent binge ethanol exposure, could impart lasting shifts in their function (Bilbo and Schwarz, 2009), perhaps priming them toward a persistent proinflammatory phenotype (Town et al., 2005; Qin et al., 2007; Qin et al., 2008; Qin and Crews, 2012; Brenhouse and Schwarz, 2016; Melbourne et al., 2021). Thus, given the vast neurodevelopmental processes occurring during adolescence, insults incurred during this critical period of neurodevelopment could have profound lasting effects on the brain that persist into adulthood and increase risk for later development of AD and AD-associated neuropathology.

Neuroinflammation and microglial activation are emerging as critical factors underlying progression of AD (Heneka et al., 2015; Dansokho and Heneka, 2018; Paudel et al., 2020), and accumulating evidence suggests neuroinflammation may even precede the onset of gross AD-associated neuropathology (Kinney et al., 2018; Long and Holtzman, 2019). In the current investigation, we report upregulation of *Hmgb1*, *Rage*, and *Tlr4* mRNA levels as well as increased expression of microglial Iba-1 and CD68 immunoreactivity in the post-mortem human basal forebrain of individuals with AUD and an adolescent age of drinking onset. HMGB1 is a ubiquitously expressed nuclear protein that is released by alcohol and other insults into the extracellular space where it acts as an endogenous agonist at RAGE and TLR4, leading to phosphorylation and nuclear translocation of NF-κB p65 and downstream induction of proinflammatory signaling molecules. Previous work from our laboratory and others report increased plasma levels of HMGB1 in adolescent binge drinkers as well as brain microglial morphological changes and upregulation of HMGB1-RAGE/TLR4-pNFκB p65 signaling in the post-mortem brain of AUD individuals with an adolescent age of drinking onset (He and Crews, 2008; Crews et al., 2013; Vetreno et al., 2013; Orio et al., 2018; Vetreno et al., 2021). HMGB1 levels are also increased in the cerebrospinal fluid of individuals with AD and associated with rapid progression of dementia (Fujita et al., 2016). Expression levels of the HMGB1 receptors RAGE and TLR4 are also increased in the post-mortem human brain of individuals with AD and positively correlated with severity of brain pathology (Lue et al., 2001; Donahue et al., 2006; Walter et al., 2007; Miron et al., 2018). Consistent with our observation of microglial morphological changes in the AUD basal forebrain, CD68-immunoreactive microglia are increased in the post-mortem basal forebrain of individuals with AD relative to controls (Wu et al., 2005). In the preclinical 5xFAD mouse model, AIE treatment accelerated induction of proinflammatory *Hmgb1*, *Rage*, *Tlr4*, *Tlr7*, *Tnfa*, and *Il6* mRNA in the adult female, but not male, basal forebrain that paralleled increased expression of microglial Iba-1, suggestive of shifts of microglia to an activated state. We have previously reported that AIE treatment in rats induces HMGB1, the HMGB1 receptors RAGE and TLR4, the nuclear transcription factor phosphorylated (activated) NFκB p65, and downstream proinflammatory cytokines throughout the brain, including the basal forebrain, that persist into adulthood (Vetreno and Crews, 2012; Vetreno et al., 2013; Vetreno et al., 2020; Crews et al., 2021). Induction of HMGB1-mediated neuroinflammation following AIE is associated with reductions of basal forebrain cholinergic neurons and cognitive deficits in adulthood (Vetreno et al., 2020; Crews et al., 2021; Crews and Vetreno, 2022; Crews et al., 2023; Macht et al., 2023). These data suggest that early life insults, such as adolescent binge drinking, increase proinflammatory signaling molecules and induce microglial morphological changes that persist into adulthood (He and Crews, 2008; Vetreno and Crews, 2012; Walter et al., 2017; Vetreno et al., 2021) that may contribute to later development of AD-associated neuropathology.

Cholinergic neurons of the basal forebrain play a major role in learning and memory through their vast projections to the hippocampus, cortex, and other brain regions (Mesulam et al., 1983; Smith and Pang, 2005). Cholinergic neurons undergo maturational refinement and consolidation of projections and receptors during adolescence (Matthews et al., 1974; Nadler et al., 1974; Zahalka et al., 1993). Multiple laboratories report that AIE, but not comparable treatment in adulthood, persistently reduces cholinergic neuron expression of ChAT, which is the enzyme responsible for ACh synthesis, in the basal forebrain of male and female mice and rats well into adulthood despite prolonged abstinence from continued alcohol exposure (Coleman et al., 2011; Ehlers et al., 2011; Vetreno et al., 2014; Swartzwelder et al., 2015; Fernandez and Savage, 2017; Vetreno and Crews, 2018). We have also reported reduced expression of ChAT and other cholinergic neuron markers in the post-mortem basal forebrain of individuals with AUD and an adolescent age of drinking onset (Vetreno et al., 2014). Reduction in basal forebrain cholinergic neuron populations is a hallmark feature of AD (Vogels et al., 1990; Cullen and Halliday, 1998; Wu et al., 2005), and incipient degeneration of basal forebrain cholinergic neurons and volumetric reductions of the basal forebrain in early adulthood and preclinical stages of AD is reported to increase risk for later development of dementia and AD (Hall et al., 2008; Teipel et al., 2011; Grothe et al., 2012; Stepanichev et al., 2017). In the current study, AIE treatment decreased ChAT+IR cholinergic neuron populations in the basal forebrain of female, but not male, 5xFAD mice. In 5xFAD mice, reductions of ChAT+ basal forebrain cholinergic neurons occur at approximately 9 months of age (Yan et al., 2018), consistent with AIE accelerating AD-associated reductions of cholinergic neuron populations. Intraneuronal accumulation of Aβ in the basal forebrain has been suggested to be an early event in the progression of AD-associated neuropathology (Arendt et al., 1988; Billings et al., 2005; LaFerla et al., 2007; Gouras et al., 2010; Baker-Nigh et al., 2015; Takahashi et al., 2017), and we report that reduction of ChAT expression in the post-mortem human basal forebrain of individuals with AUD was accompanied by increased Aβ_1–42_ immunoreactive cells, consistent with increased intraneuronal expression of Aβ_1–42_ in cholinergic neurons. Thus, these data suggest that early life insults, such as adolescent binge ethanol exposure, accelerate reductions of basal forebrain cholinergic neuron populations in female, but not male, 5xFAD mice that might contribute to later development of AD-associated dementia.

Glycyrrhizic acid, which is a constituent of *Glycyrrhiza glabra* (licorice root) classified by the FDA as Generally Recognized As Safe as a food additive, is a potent, selective HMGB1 inhibitor that binds to and inactivates released HMGB1 (Mollica et al., 2007). As HMGB1 binds to and activates RAGE and TLR4, culminating in nuclear translocation of NFκBp65 contributing to complex proinflammatory signaling (Maroso et al., 2010; Volz et al., 2010; Zurolo et al., 2011; Tang et al., 2012; Mayfield et al., 2013; Yang et al., 2020; Macht et al., 2023), HMGB1 is poised to play a crucial role as an immunoregulator underlying persistent AIE-induced proinflammatory signaling and acceleration of AD-associated neuropathology. Indeed, HMGB1-RAGE/TLR4-NF-κB p65 neuroimmune signaling in neuropathology is associated with AD and AUD (Boissiere et al., 1997; Crews et al., 2013; Vetreno et al., 2013; Fujita et al., 2016; Paudel et al., 2020; Vetreno et al., 2021). In the present study, we discovered that post-AIE administration of glycyrrhizic acid reversed persistent AIE induction of proinflammatory HMGB1 signaling, microglial activation, and persistent reduction of basal forebrain cholinergic neurons. Similar to our observations in the 5xFAD AD mouse model, glycyrrhizic acid has been reported to prevent neuroinflammation and loss of dopaminergic neurons in a rat model of Parkinson’s disease (Ojha et al., 2016). While reduction of cholinergic neuron markers is a hallmark of AD (Cullen and Halliday, 1998; Wu et al., 2005), there is little evidence of basal forebrain cholinergic neuron cell death in both clinical AD studies and preclinical AD models (Wu et al., 2005; Stepanichev et al., 2017). For instance, expression of apoptotic markers (i.e., Fas, FasL, Bax, Bcl-x, caspase 8, caspase 9, and caspase 3), when assessed by immunohistochemistry, are not present nor is there evidence of the DNA fragmentation marker TUNEL in the basal forebrain of individuals with AD (Wu et al., 2005). Interestingly, emerging studies suggest that alcohol- and proinflammatory neuroimmune-associated reduction of cholinergic neurons is not due to cell death, but rather epigenetic suppression of the cholinergic phenotype involving increased occupancy of the methylation marks RE-1 silencing transcription factor (REST) and histone 3 lysine 9 dimethylation at cholinergic phenotype gene promoters (Vetreno et al., 2020; Macht et al., 2021; Crews and Vetreno, 2022; Crews et al., 2023). Interestingly, we have reported increased REST expression in the post-mortem human basal forebrain of individuals with AUD and an adolescent age of drinking onset (Crews et al., 2023). Thus, given the reversibility of the AIE-induced acceleration of basal forebrain cholinergic neuron loss in the present study, coupled with little evidence of cell death in clinical AD studies and preclinical AD models (Wu et al., 2005; Stepanichev et al., 2017), it is plausible that targeting neuroinflammation through blockade of proinflammatory HMGB1 signaling, particularly during the early stages of AD progression, may represent a novel therapeutic approach for the treatment of AD.

In conclusion, we discovered induction of proinflammatory HMGB1-RAGE/TLR4 and evidence of microglial activation as well as increased intraneuronal Aβ_1–42_ in association with reduced cholinergic neuron marker expression (ChAT) in the post-mortem human basal forebrain of individuals with AUD relative to age-matched moderate drinking controls. Employing the preclinical 5xFAD mouse model of human AD, we discovered that AIE treatment accelerated AD-associated induction of HMGB1 proinflammatory neuroimmune genes, microglial activation, and reductions of ChAT+ basal forebrain cholinergic neurons in the adult female, but not male, basal forebrain. These preclinical findings complement our post-mortem human findings and support our hypothesis that early life adolescent binge drinking contributes to the onset and progression of AD-associated neuropathology. Our discovery that post-AIE treatment with the selective HMGB1 inhibitor glycyrrhizic acid rescues AIE-induced acceleration of AD-associated increases in proinflammatory HMGB1 neuroimmune signaling, microglial activation, and persistent reductions of basal forebrain cholinergic neurons in adult 5xFAD female mice support neuroinflammation as critical to the development and progression of AD. Together, these data suggest that adolescent binge ethanol exposure may represent an etiological risk factor contributing to onset of AD-associated neuropathology in the adult basal forebrain through HMGB1-neuroimmune signaling.

## Data Availability

The original contributions presented in this study are included in this article/Supplementary material, further inquiries can be directed to the corresponding author.
