# Supplementary material for: Adolescent binge alcohol exposure accelerates Alzheimer’s disease-associated basal forebrain neuropathology through proinflammatory HMGB1 signaling
Source: Front Aging Neurosci. 2025 Feb 19;17:1531628. doi: 10.3389/fnagi.2025.1531628 (PMC11880232; doi:10.3389/fnagi.2025.1531628)
Supplement: Supplementary file 1 [file Table_1.docx]

**
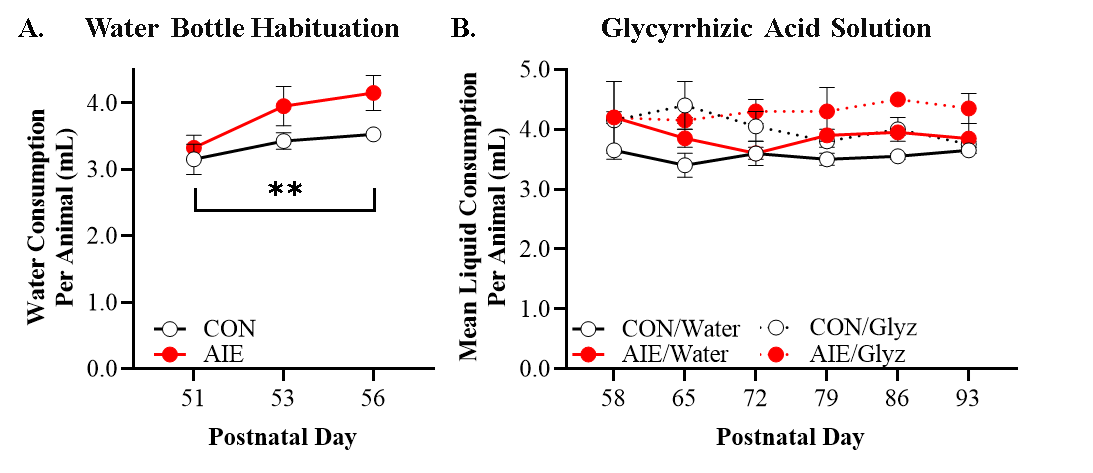
SUPPLEMENTAL FIGURE 1. Water and glycyrrhizic acid consumption during Experiment 3 in female 5xFAD mice.** **(A)** Water consumption during water bottle habituation from postnatal day (P)51 to P56. AIE treatment did not affect overall water consumption, but water consumption increased across habituation days. **(B)** During treatment of the HMGB1 inhibitor glycyrrhizic acid, subjects in the glycyrrhizic acid (Glyz) condition consumed more liquid whereas liquid consumption did not differ as a function of time or treatment condition. On average, subjects in the glycyrrhizic acid condition consumed an average of 4.0 mL/day with an approximate average consumption of 30.0 mg/kg of glycyrrhizic acid per day.

**SUPPLEMENTAL FIGURE 2. Assessment of ChAT expression in the lateral septum.**
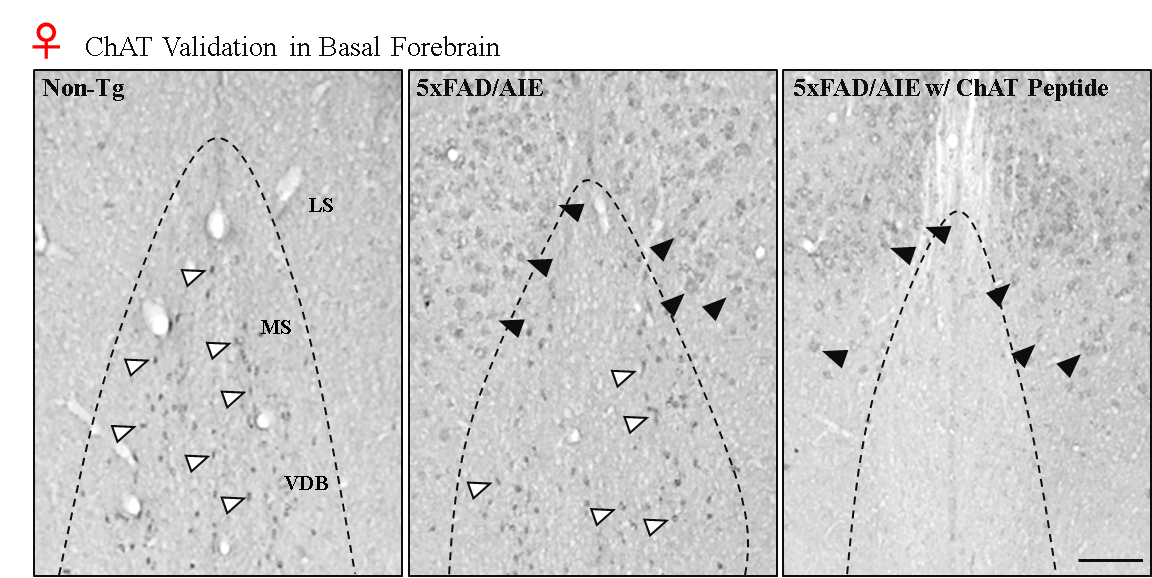
Photomicrographs depicting ChAT immunohistochemistry in the lateral septum of a non-transgenic (Non-Tg) CON female (**LEFT**), 5xFAD AIE female (**MIDDLE**), and 5xFAD AIE female wherein the ChAT antibody was preabsorbed with a ChAT blocking peptide (**RIGHT**). Notice the lack of ChAT-like plaques in the Non-Tg lateral septum whereas ChAT-like plaques are observed in 5xFAD female mice. Importantly, the staining of ChAT with a ChAT-blocking peptide blocked expression of ChAT+IR neurons (white arrows), but did not block formation of the observed ChAT-like plaques (black arrows). Thus, the plaque-like ChAT+ staining is likely a consequence of non-specific staining likely due to the “sticky” nature of amyloid plaques. LS = lateral septum, MS = medial septum, VDB = vertical limb of the diagonal band. Scale bar = 50 μm.
